# Supplementary material for: An open-source framework for large-scale, flexible evaluation of biomedical text mining systems
Source: J Biomed Discov Collab. 2008 Jan 29;3:1. doi: 10.1186/1747-5333-3-1 (PMC2276192; doi:10.1186/1747-5333-3-1)
Supplement: Additional file 1 — Evaluation platform source code. The additional file contains the software evaluation framework discussed in this paper. The most current release can be downloaded from SourceForge.net [1]. See the accompanying README file for instructions on installation and use. Also included in the distribution is a collection of UIMA wrappers for some commonly used BioNLP tools and annotated corpora. [file 1747-5333-3-1-S1.zip › bionlp_uima_repository-1.0.1/docs/api/src-html/edu/uchsc/ccp/uima/ae/nlp/parser/StanfordParser_AE.html]

```
001    /*
002     *
003     * The Original Code is UCHSC UIMA.
004     *
005     * The Initial Developer of the Original Code is University of Colorado.  
006     * Copyright (C) 2005.  All Rights Reserved.
007     *
008     * UCHSC Utilities was developed by the Center for Computational Pharmacology
009     * (http://compbio.uchsc.edu) at the University of Colorado Health 
010     *  Sciences Center School of Medicine with support from the National 
011     *  Library of Medicine.  
012     *
013     *
014     * Contributor(s):
015     *   William A. Baumgartner <william.baumgartner@uchsc.edu> (Original Author)
016     *   
017     * Created: November 2005
018     * Dependencies:
019     * TODO:
020     *      - AnnotationSet should be set dynamically. Currently it is hard-coded.
021     * Changes:
022     *      01/10/06 - wab - adjusted to conform with the updated TextAnnotation class
023     *                     - it now uses the ClassMention, SlotMention, ComplexSlotMention framework
024     *      02/08/06 - wab - output is now TokenAnnotation, PhraseAnnotation, or ClauseAnnotation
025     *      02/23/06 - wab - output now included typed dependencies, tokens are now numbered
026     *      02/26/05 - wab - Token, Phrase, and ClauseAnnotations now have proper CCPClassMentions of their own
027     *      06/14/07 - wab - If there are Sentence annotations in the CAS, then the StanfordParser is called on them, otherwise it attempts to parse the entire document text as a single sentence.
028     */
029    package edu.uchsc.ccp.uima.ae.nlp.parser;
030    
031    import java.util.ArrayList;
032    import java.util.Iterator;
033    import java.util.List;
034    
035    import org.apache.uima.analysis_engine.ResultSpecification;
036    import org.apache.uima.analysis_engine.annotator.AnnotatorConfigurationException;
037    import org.apache.uima.analysis_engine.annotator.AnnotatorContext;
038    import org.apache.uima.analysis_engine.annotator.AnnotatorContextException;
039    import org.apache.uima.analysis_engine.annotator.AnnotatorInitializationException;
040    import org.apache.uima.analysis_engine.annotator.AnnotatorProcessException;
041    import org.apache.uima.analysis_engine.annotator.JTextAnnotator_ImplBase;
042    import org.apache.uima.cas.CASException;
043    import org.apache.uima.jcas.JCas;
044    import org.apache.uima.jcas.cas.FSArray;
045    
046    import edu.uchsc.ccp.uima.annotation.CCPAnnotationSet;
047    import edu.uchsc.ccp.uima.annotation.CCPAnnotator;
048    import edu.uchsc.ccp.uima.annotation.CCPTextAnnotation;
049    import edu.uchsc.ccp.uima.annotation.syntactic.CCPClauseAnnotation;
050    import edu.uchsc.ccp.uima.annotation.syntactic.CCPPhraseAnnotation;
051    import edu.uchsc.ccp.uima.annotation.syntactic.CCPSentenceAnnotation;
052    import edu.uchsc.ccp.uima.annotation.syntactic.CCPTokenAnnotation;
053    import edu.uchsc.ccp.uima.annotation.syntactic.CCPTypedDependency;
054    import edu.uchsc.ccp.uima.mention.CCPNonComplexSlotMention;
055    import edu.uchsc.ccp.uima.mention.CCPSlotMention;
056    import edu.uchsc.ccp.uima.util.UIMA_Util;
057    import edu.uchsc.ccp.util.external.stanfordparser.InvalidTypedDependencyException;
058    import edu.uchsc.ccp.util.external.stanfordparser.StanfordParser_Util;
059    import edu.uchsc.ccp.util.external.stanfordparser.TypedDependencyCls;
060    import edu.uchsc.ccp.util.external.stanfordparser.TypedDependencyCls_Util;
061    import edu.uchsc.ccp.util.nlp.annotation.TextAnnotation;
062    import edu.uchsc.ccp.util.nlp.mention.ClassMentionTypes;
063    import edu.uchsc.ccp.util.nlp.mention.SlotMentionTypes;
064    
065    /**
066     * This class incorporates the StanfordParser into the UIMA analysis engine framework via the StanfordParserUtil.
067     * 
068     * 
069     * @author William A. Baumgartner, Jr.
070     * 
071     */
072    public class StanfordParser_AE extends JTextAnnotator_ImplBase {
073    
074        private final boolean DEBUG = false;
075    
076        private StanfordParser_Util spu;
077    
078        private final int annotatorID = 8;
079    
080        /**
081         * The location of the serialized grammar file to be used by the Stanford Parser must be included in the descriptor file under
082         * "SerializedGrammarFile"
083         */
084        public static final String PARAM_SERIALIZED_GRAMMAR_FILE = "SerializedGrammarFile";
085    
086        public static final String PARAM_ANNOTATIONSET_VALIDATOR_FILE = "AnnotationSetValidatorFile";
087    
088        public static final String PARAM_ANNOTATOR_VALIDATOR_FILE = "AnnotatorValidatorFile";
089    
090        public static final String PARAM_ATTACH_TYPED_DEPENDENCIES = "AttachTypedDependencies";
091    
092        private boolean attachTypedDependencies = false;
093    
094        /**
095         * Initializes the StanfordParser_AE by reading in the input parameters from the descriptor file and initializing a new StanfordParserUtil
096         */
097        public void initialize(AnnotatorContext ac) throws AnnotatorInitializationException, AnnotatorConfigurationException {
098            /* get input parameters from the descriptor file */
099            String serializedGrammarFile;
100            try {
101                serializedGrammarFile = (String) ac.getConfigParameterValue(PARAM_SERIALIZED_GRAMMAR_FILE);
102                attachTypedDependencies = (Boolean) ac.getConfigParameterValue(PARAM_ATTACH_TYPED_DEPENDENCIES);
103    
104            } catch (AnnotatorContextException ace) {
105                ace.printStackTrace();
106                serializedGrammarFile = null;
107            }
108    
109            /* initialize a new StanfordParserUtil */
110            spu = new StanfordParser_Util(serializedGrammarFile, attachTypedDependencies);
111    
112            super.initialize(ac);
113        }
114    
115        /**
116         * This method processes the document text, and incorporates into the CAS TokenAnnotations, PhraseAnnotations, and ClauseAnnotations based on the
117         * output of the Stanford Parser
118         */
119        public void process(JCas jcas, ResultSpecification arg1) throws AnnotatorProcessException {
120    
121            // /* get the document section IDs and lengths */
122            // Iterator it = jcas.getJFSIndexRepository().getAnnotationIndex(CCPDocumentSection.type).iterator();
123            // ArrayList<int[]> sections = new ArrayList<int[]>(); /* used for temporary storage of section id and length information */
124            // while (it.hasNext()) {
125            // /* there should be at most one CCPDocumentInformation annotation */
126            // CCPDocumentSection docSection = (CCPDocumentSection) it.next();
127            // int[] section = new int[2];
128            // section[0] = docSection.getSectionID();
129            // section[1] = docSection.getSectionLength();
130            // sections.add(section);
131            // }
132            // int[] sectionIDs = new int[sections.size()];
133            // int[] sectionLengths = new int[sections.size()];
134            // for (int i = 0; i < sections.size(); i++) {
135            // int[] section = (int[]) sections.get(0);
136            // sectionIDs[i] = section[0];
137            // sectionLengths[i] = section[1];
138            // }
139    
140            UIMA_Util uimaUtil = new UIMA_Util();
141    
142            /*
143             * Check to see if there are any Sentence annotations in this CAS. If there are, then parse each sentence individually. If there are not, then
144             * treat the document text as a single sentence and parse it.
145             */
146    
147            int numSentenceAnnotations = jcas.getJFSIndexRepository().getAnnotationIndex(CCPSentenceAnnotation.type).size();
148            if (numSentenceAnnotations > 0) {
149                Iterator sentIter = jcas.getJFSIndexRepository().getAnnotationIndex(CCPSentenceAnnotation.type).iterator();
150                while (sentIter.hasNext()) {
151                    Object possibleSent = sentIter.next();
152                    if (possibleSent instanceof CCPSentenceAnnotation) {
153                        CCPSentenceAnnotation ccpSentAnnot = (CCPSentenceAnnotation) possibleSent;
154                        String sentenceText = ccpSentAnnot.getCoveredText();
155                        int spanOffset = ccpSentAnnot.getBegin();
156                        /* parse the sentence */
157                        System.err.println("PARSING SENTENCE: " + sentenceText);
158                        List<TextAnnotation> annotations = spu.parse(sentenceText);
159                        /* correct the annotations by applying the spanOffset */
160                        for (TextAnnotation ta : annotations) {
161                            ta.setAnnotationSpanEnd(ta.getAnnotationSpanEnd() + spanOffset);
162                            ta.setAnnotationSpanStart(ta.getAnnotationSpanStart() + spanOffset);
163                        }
164                        /* add the TextAnnotations to the JCas */
165                        uimaUtil.putTextAnnotationsIntoJCas(jcas, annotations);
166                    } else {
167                        error("Expecting CCPSentenceAnnotation, but instead got: " + possibleSent.getClass().getName());
168                    }
169    
170                }
171            } else {
172                System.err.println("PARSING FULL DOCUMENT");
173                /* treat the entire document text as a single sentence */
174                /* get document text */
175                String docText = jcas.getDocumentText();
176                /* parse the text using the Stanford Parser, get back an ArrayList of TextAnnotations */
177                List<TextAnnotation> annotations = spu.parse(docText);
178                /* add the TextAnnotations to the JCas */
179                uimaUtil.putTextAnnotationsIntoJCas(jcas, annotations);
180            }
181    
182            /* now, go through the annotations, and add the more specific syntactic annotations, i.e. tokens, phrases, and clauses */
183            Iterator textAnnotIter = jcas.getJFSIndexRepository().getAnnotationIndex(CCPTextAnnotation.type).iterator();
184    
185            // int count = 0;
186            // while (textAnnotIter.hasNext()) {
187            // textAnnotIter.next();
188            // count++;
189            // }
190            // System.err.println("COUNT = " + count);
191    
192            /* initialize the annotator to use */
193            CCPAnnotator ccpAnnotator = new CCPAnnotator(jcas);
194            ccpAnnotator.setAnnotatorID(annotatorID);
195            ccpAnnotator.setFirstName("StanfordParser");
196            ccpAnnotator.setLastName("StanfordParser");
197            ccpAnnotator.setAffiliation("Stanford");
198            ccpAnnotator.addToIndexes();
199    
200            /*
201             * TODO: AnnotationSet should be set dynamically
202             * 
203             * initialize the annotation set to use
204             */
205            CCPAnnotationSet ccpAnnotationSet = new CCPAnnotationSet(jcas);
206            ccpAnnotationSet.setAnnotationSetID(7);
207            ccpAnnotationSet.setAnnotationSetName("GeneRIF Augmentation");
208            ccpAnnotationSet.setAnnotationSetDescription("Automatic augmentation of GeneRIFs with ABNER and the StanfordParser");
209            ccpAnnotationSet.addToIndexes();
210    
211            FSArray ccpAnnotationSets = new FSArray(jcas, 1);
212            ccpAnnotationSets.set(0, ccpAnnotationSet);
213    
214            List<CCPTextAnnotation> syntacticAnnotationsToRemove = new ArrayList<CCPTextAnnotation>();
215            List<CCPTextAnnotation> syntacticAnnotationsToAdd = new ArrayList<CCPTextAnnotation>();
216    
217            while (textAnnotIter.hasNext()) {
218                CCPTextAnnotation ccpTA = (CCPTextAnnotation) textAnnotIter.next();
219    
220                String mentionName = ccpTA.getClassMention().getMentionName();
221    
222                if (DEBUG) {
223                    System.out.println(ccpTA.getClassMention().getMentionName() + "; " + ccpTA.getBegin() + " -- " + ccpTA.getEnd() + ": "
224                            + ccpTA.getCoveredText());
225                }
226    
227                if (mentionName.equals(ClassMentionTypes.TOKEN)) {
228                    if (DEBUG) {
229                        System.out.print("We are converting a SyntacticAnnotation into a TokenAnnotation");
230                    }
231                    /* if it's already a TokenAnnotation, then we don't have to do anything */
232                    if (!(ccpTA instanceof CCPTokenAnnotation)) {
233                        if (DEBUG) {
234                            System.out.println("... it was not a TokenAnnotation already.");
235                        }
236                        CCPTokenAnnotation tokenAnnotation = new CCPTokenAnnotation(jcas);
237                        try {
238                            UIMA_Util.swapAnnotationInfo(ccpTA, tokenAnnotation);
239                        } catch (CASException ce) {
240                            ce.printStackTrace();
241                        }
242                        /* set the Annotator */
243                        tokenAnnotation.setAnnotator(ccpAnnotator);
244    
245                        /* set the AnnotationSet(s) */
246                        tokenAnnotation.setAnnotationSets(ccpAnnotationSets);
247    
248                        /* set POS */
249                        CCPNonComplexSlotMention ccpSlotMention = (CCPNonComplexSlotMention) UIMA_Util.getSlotMentionByName(ccpTA,
250                                SlotMentionTypes.TOKEN_PARTOFSPEECH);
251                        tokenAnnotation.setPartOfSpeech(UIMA_Util.getFirstSlotValue(ccpSlotMention));
252    
253                        /* set token number */
254                        ccpSlotMention = (CCPNonComplexSlotMention) UIMA_Util.getSlotMentionByName(ccpTA, SlotMentionTypes.TOKEN_NUMBER);
255                        tokenAnnotation.setTokenNumber(Integer.parseInt(UIMA_Util.getFirstSlotValue(ccpSlotMention)));
256    
257                        /* set typed dependencies */
258                        List<CCPSlotMention> tdSlotMentions = UIMA_Util.getMultipleSlotMentionsByName(ccpTA,
259                                SlotMentionTypes.TOKEN_TYPED_DEPENDENCY);
260    
261                        if (tdSlotMentions != null) {
262                            /* initialize a new FSArray to hold the CCPTypedDependencies */
263                            FSArray typedDependenciesArray = new FSArray(jcas, tdSlotMentions.size());
264                            for (int i = 0; i < tdSlotMentions.size(); i++) {
265                                CCPNonComplexSlotMention tdSM = (CCPNonComplexSlotMention) tdSlotMentions.get(i);
266                                String tdStr = UIMA_Util.getFirstSlotValue(tdSM);
267    
268                                TypedDependencyCls tdc;
269                                try {
270                                    tdc = TypedDependencyCls_Util.parseTypedDependencyClsString(tdStr);
271                                    CCPTypedDependency ccpTD = new CCPTypedDependency(jcas);
272                                    ccpTD.setDependentTokenNum(tdc.getDependentTokenNum());
273                                    ccpTD.setGovernorTokenNum(tdc.getGovernerTokenNum());
274                                    ccpTD.setRelation(tdc.getRelation());
275                                    ccpTD.addToIndexes();
276    
277                                    /* add it to the FSArray */
278                                    typedDependenciesArray.set(i, ccpTD);
279                                } catch (InvalidTypedDependencyException e) {
280                                    e.printStackTrace();
281                                }
282                            }
283                            /* now add the FSArray to the TokenAnnotation */
284                            tokenAnnotation.setTypedDependencies(typedDependenciesArray);
285                        }
286    
287                        // CCPComplexSlotMention csm = (CCPComplexSlotMention) UIMA_Util.getSlotMentionByName(ccpTA,
288                        // ComplexSlotMentionTypes.TOKEN_TYPED_DEPENDENCIES);
289                        // if (csm != null) {
290                        // FSArray typedDependencies = csm.getClassMentions();
291                        // /* initialize a new FSArray to hold the CCPTypedDependencies */
292                        // FSArray typedDependenciesArray = new FSArray(jcas, typedDependencies.size());
293                        // for (int i = 0; i < typedDependencies.size(); i++) {
294                        // CCPClassMention td = (CCPClassMention) typedDependencies.get(i);
295                        // /* make sure it's a typed dependency */
296                        // if (td.getMentionName().equals(ClassMentionTypes.TYPED_DEPENDENCY)) {
297                        // CCPNonComplexSlotMention ncsm = (CCPNonComplexSlotMention) UIMA_Util.getSlotMentionByName(td,
298                        // SlotMentionTypes.TYPEDEPENDENCY_GOVERNOR_TOKEN);
299                        // Integer govTokenNum = Integer.parseInt(UIMA_Util.getFirstSlotValue(ncsm));
300                        // ncsm = (CCPNonComplexSlotMention) UIMA_Util.getSlotMentionByName(td,
301                        // SlotMentionTypes.TYPEDEPENDENCY_DEPENDENT_TOKEN);
302                        // Integer depTokenNum = Integer.parseInt(UIMA_Util.getFirstSlotValue(ncsm));
303                        // ncsm = (CCPNonComplexSlotMention) UIMA_Util.getSlotMentionByName(td,
304                        // SlotMentionTypes.TYPEDEPENDENCY_RELATION);
305                        // String relation = UIMA_Util.getFirstSlotValue(ncsm);
306                        //
307                        // CCPTypedDependency ccpTD = new CCPTypedDependency(jcas);
308                        // ccpTD.setDependentTokenNum(depTokenNum);
309                        // ccpTD.setGovernorTokenNum(govTokenNum);
310                        // ccpTD.setRelation(relation);
311                        // ccpTD.addToIndexes();
312                        //
313                        // /* add it to the FSArray */
314                        // typedDependenciesArray.set(i, ccpTD);
315                        //
316                        // }
317                        // }
318                        // /* now add the FSArray to the TokenAnnotation */
319                        // tokenAnnotation.setTypedDependencies(typedDependenciesArray);
320                        // }
321    
322                        /* remove the old annotation from the JCas, and add the new one */
323                        // ccpTA.removeFromIndexes();
324                        syntacticAnnotationsToRemove.add(ccpTA);
325                        syntacticAnnotationsToAdd.add(tokenAnnotation);
326                        // tokenAnnotation.addToIndexes();
327    
328                    }
329    
330                } else if (mentionName.equals(ClassMentionTypes.PHRASE)) {
331                    /* if it's already a PhraseAnnotation, then we don't have to do anything */
332                    if (!(ccpTA instanceof CCPPhraseAnnotation)) {
333                        CCPPhraseAnnotation phraseAnnotation = new CCPPhraseAnnotation(jcas);
334                        try {
335                            UIMA_Util.swapAnnotationInfo(ccpTA, phraseAnnotation);
336                        } catch (CASException ce) {
337                            ce.printStackTrace();
338                        }
339                        /* set the Annotator */
340                        phraseAnnotation.setAnnotator(ccpAnnotator);
341    
342                        /* set the AnnotationSet(s) */
343                        phraseAnnotation.setAnnotationSets(ccpAnnotationSets);
344    
345                        /* set the PhraseType */
346                        CCPNonComplexSlotMention ccpSlotMention = (CCPNonComplexSlotMention) UIMA_Util.getSlotMentionByName(ccpTA,
347                                SlotMentionTypes.PHRASE_TYPE);
348                        phraseAnnotation.setPhraseType(UIMA_Util.getFirstSlotValue(ccpSlotMention));
349    
350                        /* remove the old annotation from the JCas, and add the new one */
351                        // ccpTA.removeFromIndexes();
352                        syntacticAnnotationsToRemove.add(ccpTA);
353                        // phraseAnnotation.addToIndexes();
354                        syntacticAnnotationsToAdd.add(phraseAnnotation);
355                    }
356                } else if (mentionName.equals(ClassMentionTypes.CLAUSE)) {
357                    /* if it's already a ClauseAnnotation, then we don't have to do anything */
358                    if (!(ccpTA instanceof CCPClauseAnnotation)) {
359                        CCPClauseAnnotation clauseAnnotation = new CCPClauseAnnotation(jcas);
360                        try {
361                            UIMA_Util.swapAnnotationInfo(ccpTA, clauseAnnotation);
362                        } catch (CASException ce) {
363                            ce.printStackTrace();
364                        }
365    
366                        /* set the Annotator */
367                        clauseAnnotation.setAnnotator(ccpAnnotator);
368    
369                        /* set the AnnotationSet(s) */
370                        clauseAnnotation.setAnnotationSets(ccpAnnotationSets);
371    
372                        /* set the clauseType */
373                        CCPNonComplexSlotMention ccpSlotMention = (CCPNonComplexSlotMention) UIMA_Util.getSlotMentionByName(ccpTA,
374                                SlotMentionTypes.CLAUSE_TYPE);
375                        clauseAnnotation.setClauseType(UIMA_Util.getFirstSlotValue(ccpSlotMention));
376    
377                        /* remove the old annotation from the JCas, and add the new one */
378                        // ccpTA.removeFromIndexes();
379                        syntacticAnnotationsToRemove.add(ccpTA);
380                        // clauseAnnotation.addToIndexes();
381                        syntacticAnnotationsToAdd.add(clauseAnnotation);
382                    }
383                } else {
384                    /* do nothing with anything other than token, phrase, or clause */
385                }
386    
387            }
388    
389            /* add the new syntactic annotations (must add outside of while loop to avoid concurrent modification exception */
390            for (CCPTextAnnotation ccpTA : syntacticAnnotationsToAdd) {
391                ccpTA.addToIndexes();
392            }
393    
394            /* Removed the now unnecessary SyntacticAnnotations */
395            for (CCPTextAnnotation ccpTA : syntacticAnnotationsToRemove) {
396                ccpTA.removeFromIndexes();
397            }
398        }
399    
400        private void error(String message) {
401            System.err.println("ERROR -- StanfordParser_AE: " + message);
402        }
403    
404    }
405    // // for each returned TextAnnotation, create a new TokenAnnotation
406    // for (Iterator iter = tokens.iterator(); iter.hasNext();) {
407    // TextAnnotation ta = (TextAnnotation) iter.next();
408    //
409    // Annotator annotator = new Annotator(new Integer(annotatorID), "StanfordParser", "StanfordParser", "Stanford");
410    // ta.setAnnotator(annotator);
411    //
412    // // The Annotation Set should be dynamically set in the future.
413    // AnnotationSet annotationSet = new AnnotationSet(new Integer(7), "GeneRIF Augmentation",
414    // "Automatic augmentation of GeneRIFs with ABNER and the StanfordParser");
415    // List<AnnotationSet> annotationSets = new ArrayList<AnnotationSet>();
416    // annotationSets.add(annotationSet);
417    // ta.setAnnotationSets(annotationSets);
418    //
419    // String mentionType = (String) ta.getClassMention().getMentionName();
420    //
421    // SyntacticAnnotation syntacticAnnotation;
422    //
423    // if (mentionType.equalsIgnoreCase(ClassMentionTypes.TOKEN)) {
424    // // create a new TokenAnnotation
425    // syntacticAnnotation = new TokenAnnotation(jcas);
426    //
427    // // set POS
428    // SlotMention slotMention = ta.getClassMention().getSlotMentionByName(SlotMentionTypes.TOKEN_PARTOFSPEECH);
429    // ((TokenAnnotation) syntacticAnnotation).setPartOfSpeech((String) slotMention.getFirstSlotValue());
430    //
431    // // set token number
432    // slotMention = ta.getClassMention().getSlotMentionByName(SlotMentionTypes.TOKEN_NUMBER);
433    // ((TokenAnnotation) syntacticAnnotation).setTokenNumber(((Integer) slotMention.getFirstSlotValue()));
434    //
435    // // set typed dependencies
436    // ComplexSlotMention csm = ta.getClassMention().getComplexSlotMentionByName(ComplexSlotMentionTypes.TOKEN_TYPED_DEPENDENCIES);
437    // if (csm != null) {
438    // List<ClassMention> typedDependencies = csm.getClassMentions();
439    // // initialize a new FSArray to hold the CCPTypedDependencies
440    // FSArray typedDependenciesArray = new FSArray(jcas, typedDependencies.size());
441    // for (int i = 0; i < typedDependencies.size(); i++) {
442    // ClassMention td = typedDependencies.get(i);
443    // // make sure it's a typed dependency
444    // if (td.getMentionName().equals(ClassMentionTypes.TYPED_DEPENDENCY)) {
445    // Integer govTokenNum = (Integer) td.getSlotMentionByName(SlotMentionTypes.TYPEDEPENDENCY_GOVERNOR_TOKEN)
446    // .getFirstSlotValue();
447    // Integer depTokenNum = (Integer) td.getSlotMentionByName(SlotMentionTypes.TYPEDEPENDENCY_DEPENDENT_TOKEN)
448    // .getFirstSlotValue();
449    // String relation = (String) td.getSlotMentionByName(SlotMentionTypes.TYPEDEPENDENCY_RELATION)
450    // .getFirstSlotValue();
451    //
452    // CCPTypedDependency ccpTD = new CCPTypedDependency(jcas);
453    // ccpTD.setDependentTokenNum(depTokenNum);
454    // ccpTD.setGovernorTokenNum(govTokenNum);
455    // ccpTD.setRelation(relation);
456    // ccpTD.addToIndexes();
457    //
458    // // add it to the FSArray
459    // typedDependenciesArray.set(i, ccpTD);
460    //
461    // }
462    // }
463    // // now add the FSArray to the TokenAnnotation
464    // ((TokenAnnotation) syntacticAnnotation).setTypedDependencies(typedDependenciesArray);
465    // }
466    //
467    // } else if (mentionType.equalsIgnoreCase(ClassMentionTypes.PHRASE)) {
468    // // create a new PhraseAnnotation
469    // syntacticAnnotation = new PhraseAnnotation(jcas);
470    // SlotMention slotMention = ta.getClassMention().getSlotMentionByName(SlotMentionTypes.PHRASE_TYPE);
471    // ((PhraseAnnotation) syntacticAnnotation).setPhraseType((String) slotMention.getFirstSlotValue());
472    // } else if (mentionType.equalsIgnoreCase(ClassMentionTypes.CLAUSE)) {
473    // // create a new ClauseAnnotation
474    // syntacticAnnotation = new ClauseAnnotation(jcas);
475    // SlotMention slotMention = ta.getClassMention().getSlotMentionByName(SlotMentionTypes.CLAUSE_TYPE);
476    // ((ClauseAnnotation) syntacticAnnotation).setClauseType((String) slotMention.getFirstSlotValue());
477    // } else if (mentionType.equalsIgnoreCase(ClassMentionTypes.SENTENCE)) {
478    // // create a new SentenceAnnotation
479    // // This currently 2/23/06 does not output any sentences.
480    // // Instead, S, is output as a clause
481    // syntacticAnnotation = new SentenceAnnotation(jcas);
482    // } else {
483    // // should never be here, but just in case, create a base
484    // // SyntacticAnnotation
485    // syntacticAnnotation = new SyntacticAnnotation(jcas);
486    // System.err.println("A base syntactic annotation was created by the StanfordParser_AE. This should never occur.");
487    // }
488    //
489    // UIMA_Util.swapAnnotationInfo(ta, syntacticAnnotation, jcas);
490    //
491    // /*
492    // * create a new CCPClassMention - this is somewhat redundant since
493    // * we know this is already a Token, however it allows the annotation
494    // * to be used by tools which only use the more general
495    // * semantic/syntactic annotations within the UIMA framework
496    // */
497    // // init a new CCPClassMention
498    // CCPClassMention ccpClassMention = new CCPClassMention(jcas);
499    // ccpClassMention.setMentionName(mentionType);
500    //
501    // // get the slot mentions to be put into the ccpClassMention
502    // Collection<SlotMention> slotMentions = ta.getClassMention().getSlotMentions();
503    // Collection<ComplexSlotMention> complexSlotMentions = ta.getClassMention().getComplexSlotMentions();
504    //
505    // // init the array that will hold the ccp mentions
506    // FSArray ccpSlotMentionsArray = new FSArray(jcas, slotMentions.size() + complexSlotMentions.size());
507    // int mentionIndex = 0;
508    //
509    // // for each slot mention, add it to the ccpSlotMentionsArray
510    // for (SlotMention sm : slotMentions) {
511    // CCPNonComplexSlotMention ccpNonComplexSlotMention = new CCPNonComplexSlotMention(jcas);
512    // ccpNonComplexSlotMention.setMentionName(sm.getMentionName());
513    //
514    // // transfer the slot values
515    // List<Object> slotValues = sm.getSlotValues();
516    // StringArray slotValuesArray = new StringArray(jcas, slotValues.size());
517    // for (int i = 0; i < slotValues.size(); i++) {
518    // Object value = slotValues.get(i);
519    // if (value instanceof Integer) {
520    // slotValuesArray.set(i, ((Integer) value).toString());
521    // } else {
522    // // assume it's a String
523    // slotValuesArray.set(i, (String) value);
524    // }
525    // }
526    // ccpNonComplexSlotMention.setSlotValues(slotValuesArray);
527    // ccpSlotMentionsArray.set(mentionIndex++, ccpNonComplexSlotMention);
528    // }
529    //
530    // // for each complex slot mention, add it to the ccpSlotMentionsArray
531    // for (ComplexSlotMention csm : complexSlotMentions) {
532    // // init a new CCPComplexSlotMention
533    // CCPComplexSlotMention ccpComplexSlotMention = new CCPComplexSlotMention(jcas);
534    // ccpComplexSlotMention.setMentionName(csm.getMentionName());
535    //
536    // //
537    // }
538    //
539    // // if it is not a SentenceAnnotation then add the appropriate slot
540    // if (!mentionType.equals(ClassMentionTypes.SENTENCE)) {
541    // String slotName = ta.getClassMention().getFirstSlotMention().getMentionName();
542    // String label = (String) ta.getClassMention().getFirstSlotMention().getFirstSlotValue();
543    // try {
544    // UIMA_Util.addSlotValue(ccpClassMention, slotName, label);
545    // } catch (CASException e) {
546    // e.printStackTrace();
547    // }
548    // }
549    // syntacticAnnotation.setClassMention(ccpClassMention);
550    // if (sectionIDs.length > 0) {
551    // syntacticAnnotation.setDocumentSectionID(GenericTextUtils.determineDocumentSection(ta.getFirstSpanStartIndex(), sectionIDs,
552    // sectionLengths));
553    // }
554    // syntacticAnnotation.addToIndexes();
555    //
556    // }
557    // }
558    // }
```
